# Recent Workmen's Compensation Cases

**Published:** 1910-02-26

**Authors:** 


### SPECIAL ARTICLE.

### RECENT WORKMEN'S COMPENSATION CASES.

A sidelight was thrown upon the working of the Act during a case heard by Judge Shaw at Liverpool on October 29. A man who had been injured was paid compensation for a considerable time, after which the employers sought to reduce it on the ground that he was able to resume his ordinary employment. Judge Shaw, who held that he was not fit for his regular work, said: "There is no question of whether he is able to get light work from anybody else. I know as a matter of fact that he cannot. I do not have five or six of these cases before me nearly every day without knowing that a man who is deficient in any small particular does not meet with work at all."

In a case heard in the Court of Appeal on November 10 the question was raised as to whether a one-eyed collier, having a perfectly sound eye, could be said to be disabled. It appeared that in 1905 he met with an accident by which one of his eyes was completely destroyed. Compensation was paid for a long time, but on January 30 the employers alleged that he was able to resume his full work as a collier. The County Court Judge held, as a fact, that he was able to earn his full wages. In the Court of Appeal it was concluded that through the loss of an eye the man was exposed to more than ordinary risk as a collier. If a man with both eyes should get dust in an eye he had still one to see with; but in this case he would probably be blind under such circumstances. The Court held that it was a matter of fact for the County Court Judge to decide, and would not interfere.

At Epping County Court an application was made by the widow of a gardener who met with his death in the following circumstances: It was part of his duty to look after fowls. Towards the end of 1908 a young cockerel pecked him in the arm, in consequence of which blood-poisoning supervened. About a month later symptoms of insanity were shown, and the unfortunate man whilst in this condition committted suicide. It was held that these results were the sequelæ of the accident, and the widow was awarded £156, with costs.

In the Court of Session, October 31, a workman who was in receipt of compensation had it stopped on the ground that he was no longer incapacitated. The employers and the workman then both applied that the matter might be referred to a medical referee, who reported that the incapacity ceased on October 20, 1908. In May 1909 the workman applied for renewal of the compensation. It was held that the report of the referee was no bar to the claim. In a case at Bradford (November 2) a claim was made by a mill hand for half wages on account of disablement by anthrax. She was employed at a combing mill. She left her work, feeling unwell, on January 27, and subsequently a pustule was found to have formed under her chin. The Judge said he had to look for the most probable origin of the disease. It was material to notice that by Section 8 (2) of the Act an industrial disease is deemed to have been due to the nature of the employment unless the employer proves the contrary. Here the employer called no evidence, and certainly did not prove the contrary. In the result, the employers were held liable.

### Incapacity After Accident.

Workmen who are receiving partial compensation and doing light work sometimes imagine that if the work ceases, even through their own misconduct, they are entitled to a full award. This is not so. In a recent case in the Court of Appeal it appeared that the applicant had all the forefingers of one hand twisted by an accident. Full compensation was paid him for a considerable time, and then his employers found him a position superior to that which he formerly held, in which he could earn more wages. On being dismissed for drunkenness, he applied for a renewal of the compensation. This the Court of Appeal refused to allow, without prejudice, however, to another application.

### Alleged Lead-poisoning.

One usually associates lead-poisoning with those who toil in glassworks and other places where oxide of lead comes into the manufacture. In a case recently heard at Birmingham, the applicant was employed by the respondent at a "press-on" lamp work. In two days she handled thirteen gross of the lamps, the edges of which had been fastened with solder. The process she was engaged in involved handling the lamps three times. She got into a habit of wetting her fingers in order to facilitate picking up. Subsequently she developed lead-poisoning, and ceased work on May 13, after obtaining a certificate from the factory doctor. She was not aware of any other cause for her illness. Mr. L. Gamgee spoke to examining applicant in October. She was then suffering from lead-poisoning. He had made himself familiar with the process she had been engaged in, and he did not think she could have contracted the disease in that way. Dr. Haines said applicant was suffering from lead-poisoning on April 29, and he considered her condition then was consistent with her having been given to the habit referred to; but he could not remember, in his own experience, an authoritative case of poisoning from metallic lead. His Honour said he was not satisfied that the illness arose owing to the nature of the employment, and judgment must be for the respondents.

### Accident or Tobacco-poisoning.

In a case heard at Liverpool (December 8) it appeared that an award had been made on April 21 in favour of a glass-blower who was struck in the eye by a piece of hot glass on November 30, 1908. Shortly afterwards he was examined by Dr. Kerr, who found an external abrasion, but no injury to the eye itself. On December 3 the same doctor found he was suffering from hypermetropia. On December 22 Dr. Jones formed the opinion that the then defective condition of the right eye was much older than the date of the injury. In March 1909, in consequence of a report that the man was suffering from iritis, an award was made, with the proviso that it should be reduced by one-half if the applicant took up light work. He went back to the glass furnace, but gave up working there on the ground that he could not stand the glare. On an application to review this award, his Honour Judge Shand sat with Dr. Shears as medical referee. A number of medical witnesses were called. It transpired that there had been a remarkable depreciation in the applicant's eyesight since July 25, 1909. Dr. Edgar Browne said he found that with his right eye Roberts (the applicant) could not see a hand six inches away, and in the left eye he found traces of tobacco-poisoning. The applicant smoked half an ounce of twist or black cavendish a day. That was the tobacco which caused most of the tobacco-poisoning in the North of England. Tobacco-poisoning could be cured by three months' abstinence. Other witnesses stated that the man's eyes would be affected by standing a long time before a furnace. In the event the judge and the referee retired with the applicant. On returning to Court the judge said that he had now recovered from the effects of the accident. His present condition was due to tobacco-poisoning, and it was clear that hypermetropia in both eyes existed prior to the accident. The award was terminated.

### Accident or Apoplexy.

In a case at Wrexham the question to decide was whether applicant's husband died from natural causes or as the result of an accident. The applicant stated that her husband was working in the mine on the morning of September 6, and was found by his mate vomiting, and lying on the ground. He had a swelling on the back of the head and looked as if he had sustained a blow. The respondents' case was that the man died as the result of an apoplectic seizure. Dr. D. J. Williams, Rhos, said he did not think death followed on an apoplectic seizure, but was the result of a blow on the back of the head. The organs, blood-vessels, and arteries were healthy. Dr. J. C. Davies, Rhos, said he was present at the post-mortem examination conducted by the last witness, and he thought death was due to apoplexy, and not to any injury. The veins and arteries were diseased. Dr. Richard Williams (Wrexham) and Dr. Hislop confirmed the opinion of the last witness. His Honour took time to consider the case, and, in giving judgment, he said the case was a somewhat curious one and not very easy to decide. In the course of the evidence there was a deplorable conflict in the medical evidence. That being so, the burden of proof rested affirmatively upon the applicant. He awarded the applicant £274 7s. 1d., to be distributed and paid for the benefit of her dependents at the rate of £2 a month.

A case was heard recently at the Mansfield County Court in which a miner employed at the Bentinck Colliery claimed from the New Hucknall Colliery Company the sum of £2 as compensation for injuries said to be due to the sting of an insect. It appeared that he was working in a hot part of the pit and was stung in the forehead by some unknown insect. Blood-poisoning supervened, and he was too ill to work for a fortnight. The evidence showed that in the place in question, which was the last to receive ventilation, the great heat causes gnats, beetles, hornets, and flies of different sorts to congregate. It also appeared that the air was foul, and that refuse was unavoidably left there, which rapidly decomposed in the heat, and also that the ponies in the pit sometimes had sores, on which flies and insects settled. His Honour held that the accident arose out of and in the course of the employment, and awarded compensation.

At a meeting of an insurance and actuarial society in Glasgow on December 6 a paper was read by Dr. Arthur Mechan on "Some medical aspects of the Workmen's Compensation Acts in their relation to insurance." Dr. Mechan pointed out that the Acts of 1897 and 1906, though apparently simple to read and easy of construction, avowedly designed, moreover, to do away with litigation, had, in fact, led to more litigation and medical controversy than any other enactment of modern times. He referred to the progressive increase in claims and the certainty that the tendency to increase had by no means reached its limit, particularly in the case of industrial diseases, in connection with which medical men had been unable to restrict unwise extension. Touching on what he called extraordinary decisions that had been given in reference to the acceleration of death or disease due to accident, and the increasing difficulties of dealing with this feature, he said that an increased knowledge of the subject was being acquired by claimants, or rather their law agents. In this connection he considered Glasgow was ingloriously prominent in the possession of agents who were quite ready to make unscrupulous use of the Compensation Acts.

### Medical Evidence.

A prominent feature of the cases which so often occur in the county courts in relation to workmen's compensation is that they appear to turn more and more on purely medical points. So often is this the case that the County Court judge is frequently compelled to employ a medical referee with whom the decision of the case is practically allowed to rest. The medical practitioner, too, is practically the sole arbiter of the questions—(*a*) What is an accident? and (*b*) Is this man still incapacitated from work?

In a case at Barnsley, on January 13, it appeared that a collier was struck in the eye by a piece of coal. The result of the accident was that the injured eye deceived him when looking at an object such as a candle. He refused to work at the coal face in this condition. The employers refused to find him work on the surface. "The question I am asked to decide," said his Honour Judge Dodd, "is whether getting coal at the coal face is a suitable employment for a miner who has recently had one of his eyes injured by an accident whilst so employed. I am of opinion that it is not quite suitable employment for a man circumstanced as the applicant is."

It appeared, in another case, that the applicant's husband, while doing the ordinary work of a collier, died quite suddenly. The post-mortem disclosed the fact that the right auricle of the heart was ruptured, and that such rupture was the cause of death. The doctors differed on the question whether the heart was normal or not. Judge Bryn Roberts, in giving judgment, said it seemed to him to be immaterial whether the deceased had a sound or a diseased heart, provided that death resulted from a strain received while the workman was at work. It did not matter whether such strain was of a kind usually accompanying the work in which the workman was engaged or not. An award of £272 was made to the widow.

Although it is not strictly a compensation case, mention may be made of an inquest which was held on January 7 at Sheffield on the body of a miner named Gascoigne. It was alleged that he died of blood poisoning due to an injury which he sustained in the pit. It appeared that his hand had been crushed between a prop and a tub. A juror remarked that if "cartgrease" got into the wound it would set up blood poisoning. There was a conflict of evidence as to whether this could have caused the blood poisoning of which the man died. The jury returned a verdict that the man died of blood poisoning caused by an injury to his hand whilst at work in the pit.

In a case heard at Newcastle on December 31 it appeared that on December 27, 1908, the applicant was employed as second engineer on a steamer on the River Plate. On the day in question the engine room was ill-ventilated, and the temperature was 117 degrees. While at his work the applicant ruptured a blood-vessel in the brain and subsequently became completely paralysed. The question was whether this was caused by the applicant working in a temperature of 117 degrees, and whether it was an accident within the meaning of the Act. Medical evidence was called on both sides, the witnesses for the respondent stating that they were of opinion that the paralysis from which the applicant was suffering was due to natural causes, and was not the result of the condition under which he was working at the time. In giving judgment his Honour said that if he could properly come to the conclusion that the doctors at the River Plate were right in saying that the man suffered from heatstroke, then it was impossible for him to say that it was not an accident. An award was made, but a stay of execution was granted pending an appeal.

